# The role of left ventricular deformation in the assessment of microvascular obstruction and intramyocardial haemorrhage

**DOI:** 10.1007/s10554-016-1006-x

**Published:** 2016-10-26

**Authors:** Pankaj Garg, Ananth Kidambi, Peter P. Swoboda, James R. J. Foley, Tarique A. Musa, David P. Ripley, Bara Erhayiem, Laura E. Dobson, Adam K. McDiarmid, Graham J. Fent, Philip Haaf, John P. Greenwood, Sven Plein

**Affiliations:** 1 Multidisciplinary Cardiovascular Research Centre and Division of Biomedical Imaging, Leeds Institute of Cardiovascular and Metabolic Medicine, University of Leeds, Leeds, UK; 2 Department of Cardiology and Cardiovascular Research Institute Basel (CRIB), University Hospital Basel, Basel, Switzerland

**Keywords:** Haemorrhage, Cardiovascular magnetic resonance, Myocardial infarction, Left ventricular function

## Abstract

In the setting of acute ST-elevation myocardial infarction (STEMI), it remains unclear which strain parameter most strongly correlates with microvascular obstruction (MVO) or intramyocardial haemorrhage (IMH). We aimed to investigate the association of MVO, IMH and convalescent left ventricular (LV) remodelling with strain parameters measured with cardiovascular magnetic resonance (CMR). Forty-three patients with reperfused STEMI and 10 age and gender matched healthy controls underwent CMR within 3-days and at 3-months following reperfused STEMI. Cine, T2-weighted, T2*-imaging and late gadolinium enhancement (LGE) imaging were performed. Infarct size, MVO and IMH were quantified. Peak global longitudinal strain (GLS), global radial strain (GRS), global circumferential strain (GCS) and their strain rates were derived by feature tracking analysis of LV short-axis, 4-chamber and 2-chamber cines. All 43 patients and ten controls completed the baseline scan and 34 patients completed 3-month scans. In multivariate regression, GLS demonstrated the strongest association with MVO or IMH (beta = 0.53, p < 0.001). The optimal cut-off value for GLS was −13.7% for the detection of MVO or IMH (sensitivity 76% and specificity 77.8%). At follow up, 17% (n = 6) of patients had adverse LV remodeling (defined as an absolute increase of LV end-diastolic/end-systolic volumes >20%). Baseline GLS also demonstrated the strongest diagnostic performance in predicting adverse LV remodelling (AUC = 0.79; 95% CI 0.60–0.98; p = 0.03). Post-reperfused STEMI, baseline GLS was most closely associated with the presence of MVO or IMH. Baseline GLS was more strongly associated with adverse LV remodelling than other CMR parameters.

## Introduction

Microvascular obstruction (MVO) and intra-myocardial haemorrhage (IMH) as detected by cardiovascular magnetic resonance (CMR) are established independent adverse prognostic markers following reperfused ST-elevation myocardial infarction (STEMI). The presence of MVO has been associated with ‘no re-flow’ on coronary angiography after revascularisation [1]. IMH is invariably associated with MVO and is caused by endothelial dysfunction following prolonged ischaemia/reperfusion injury with disruption of inter-endothelial junctions and extravasation of erythrocytes [2].

Myocardial systolic function after STEMI is conventionally assessed by calculating left ventricular ejection fraction (EF) from left ventricular volumes [3–5]. However, global EF is load-dependent and neglects regional function [6]. Myocardial deformation may be a more accurate parameter of LV function, but its assessment is more challenging, due in part to the complex spatial orientation and distribution of muscle fibres in the longitudinal and circumferential direction [7]. Emerging technologies have made it possible to study myocardial deformation by CMR using myocardial tagging and feature tracking (FT) derived strain [8, 9]. Strain (S) and strain rate (SR) are already established as more accurate measures of both regional and the global left ventricular function when compared to ejection fraction and allow quantitative assessment of myocardial deformation [10]. From strain analysis, several parameters can be derived and it is currently not known which of these, if any, are associated with the presence of MVO, IMH and adverse LV remodelling.

This study aimed to investigate the association of FT derived peak global longitudinal strain (GLS), peak global circumferential strain (GCS), peak global radial strain (GRS), peak global longitudinal strain rate (GLSR), peak global circumferential strain rate (GCSR) and peak global radial strain rate (GRSR) with the presence of MVO, IMH and adverse LV remodelling in acute reperfused STEMI.

## Methods

### Study population

**Fig. 1:**
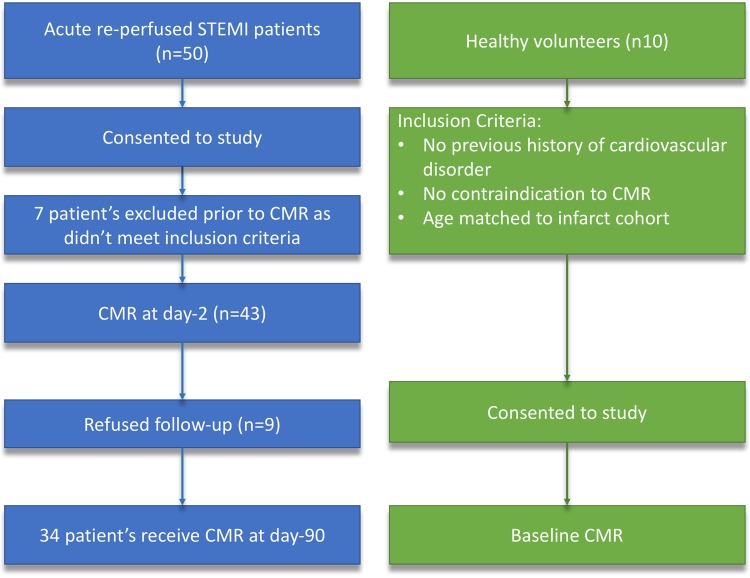
Flow chart of the study cohort

Fifty-three subjects were prospectively recruited from a single large UK tertiary centre. They included forty-three patients with acute STEMI and ten age and sex matched healthy volunteers serving as controls (Fig. 1). The inclusion criteria for STEMI patients were: first-time acute STEMI revascularized by primary percutaneous coronary intervention (PPCI) within 12 h of onset of chest pain. Acute STEMI was defined as per the current European Society of Cardiology (ESC) guidelines [11]. Exclusion criteria included: previous MI or coronary artery bypass grafting, cardiomyopathy, estimated glomerular filtration rate <30 ml/min/1.73 m^2^, haemodynamic instability (Killip class III/IV requiring on-going intravenous therapy [12]) and contraindication to CMR imaging. After PPCI, all patients were considered for ESC guideline approved post-myocardial infarction secondary prevention therapy at the discretion of the treating physician, and were enrolled in a cardiac rehabilitation programme if they were deemed suitable [11]. Healthy volunteers had no history or symptoms of cardiovascular disease, were on no cardiovascular or other relevant medication and had no contraindications to CMR.

### Ethics approval

The study protocol was approved by the National Research Ethics Service (12/YH/0169) and complied with the Declaration of Helsinki and all patients gave written informed consent.

### Cardiac catheterization

Coronary angiography and revascularisation were performed in a standard fashion as per current best practice guidelines [13]. TIMI flow grades were assessed visually as described previously after coronary angioplasty [21].

### CMR examination

All patients underwent CMR imaging at 3.0 T (Achieva TX, Philips Healthcare, Best, The Netherlands) within 3 days (median 2 days) of their index presentation and were invited to attend a further CMR study at 3 months. CMR imaging used a dedicated 32-channel cardiac phased array receiver coil. Cine imaging was performed using a balanced steady-state free precession (SSFP) pulse sequence with a spatial resolution of 1.6 × 2.0 × 10 mm and 40 phases per cardiac cycle. 4-chamber, 2-chamber and LV short axis stack cine imaging were acquired for strain analysis using the same spatial and temporal resolution.

T2 weighted (T2w) and T2* imaging were performed using the ‘3-of-5’ approach by acquiring the central 3 slices of 5 parallel short-axis slices spaced equally from mitral valve annulus to LV apical cap [14]. 0.1 mmol/kg gadolinium-DTPA (gadopentetate dimeglumine; Magnevist, Bayer, Berlin, Germany) was administered using a power injector (Spectris, Solaris, PA). Late gadolinium enhancement (LGE) was performed in 10–12 short-axis slices 16–20 min after contrast administration using an inversion recovery-prepared T1-weighted gradient echo-pulse sequence. For each pulse sequence, images with artefact were repeated until any artefact was removed or minimized. The highest quality images were used for analysis.

### Image analysis

Cine, T2w, T2* and LGE images were evaluated offline using commercially available software (cvi42 v5.1, Circle Cardiovascular Imaging Inc., Calgary, Canada). Left ventricular volumes and EF were analyzed from cine images using standard methods [15]. Infarct location was determined by LGE imaging, according to standard guidelines [16]. The presence and size of infarction and MVO were measured from LGE images. Infarcted myocardium was defined as an area of LGE ≥ 2 standard deviations (SD) above remote myocardium, and infarct volume estimation included any hypointense core. We used the 2SD method as there are prognostic data for the 2SD infarct size estimation in similar populations [17], and for consistency with analysis of T2w images. MVO was defined visually as the hypointense core within the infarcted zone and planimetered manually. Volumes of infarct and MVO were calculated from planimetered areas through the whole short-axis LV LGE stack by the modified Simpson’s method. The presence and extent of intra-myocardial haemorrhage was assessed by combined analysis of T2w and T2* sequences [8]. On T2w images, areas with mean signal intensity less than 2 SD below the periphery of the area at risk (AAR) were considered to be haemorrhage [18]. On the T2* images, the presence of a dark core within the infarcted area by visual inspection of the images was used as confirmation of myocardial haemorrhage. Concordant results between T2w and T2* were needed to confirm haemorrhage. If there was inconsistency between them, agreement between two experts informed the results. Presence/absence of both MVO and IMH were scored in a binary manner.

### Strain analysis

**Fig. 2:**
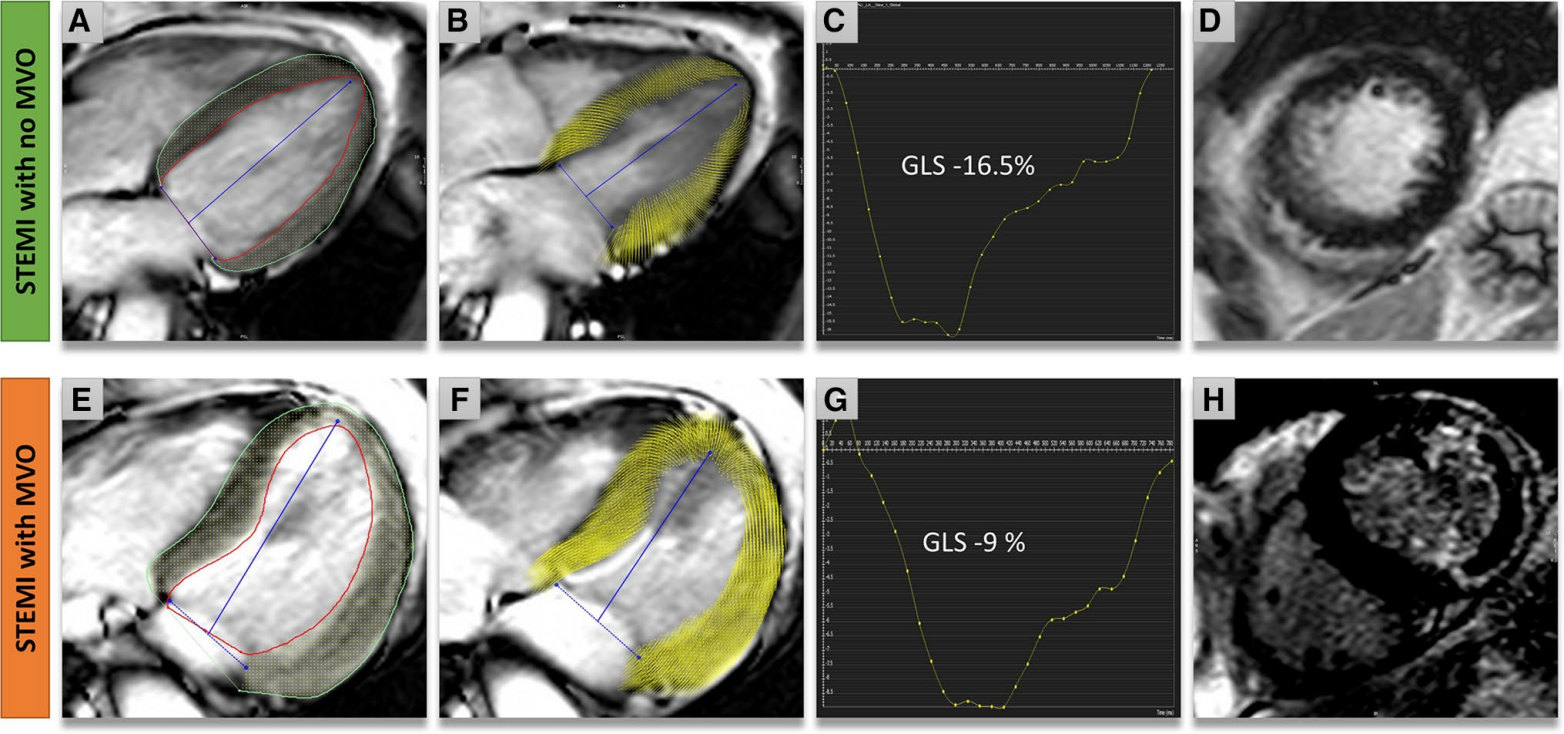
Multi-parametric CMR examination of two patients with ST-elevation myocardial infarction. Case 1 (**a**–**d**): Anterior MI without MVO. **a** Epicardial (*green*) and endocardial (*red*) contours on a 4-chamber cine. **b** Voxel derived feature tracking (FT) of the myocardium at end-systole. **c** Global longitudinal strain (GLS) *curve* demonstrating a GLS of −16.5%. **d** LGE short-axis demonstrating infarct in anterior wall. Case 2 (**e**–**h**): **e** Demonstrates the contours and (**f**) shows the end-systolic FT-derived strain myocardial points in a case of lateral infarction with MVO. **g** Demonstrates a significantly lower GLS, −9%. **h** Demonstrates infarct and presence of MVO on LGE-images

Strain analysis was performed in a semi-automated manner using Circle Cardiovascular Imaging Inc., Calgary, Canada cvi42 v5.1 (Fig. 2). The observer performing the strain analysis was blinded to the baseline CMR parameters and advanced tissue characterization. Left ventricular endocardial and epicardial borders were manually contoured in end-diastole from both long-axis cines (4-chamber and 2 chamber). Endocardial borders, epicardial borders and reference points at both RV insertion points (anterior/inferior) were contoured manually for each slice at end-diastole from the short axis LV cine stack. GLS and GLSR were derived from the long-axis images and GRS, GRSR, GCS and GCSR were derived from the short-axis LV cine stack using published methods [19, 20]. Peak GLS, peak GLSR, GRS, peak GRSR, peak GCS and peak GCSR were quantified.

### Follow-up scans

Follow-up scans were planned at 3 months following the index event. Patients were divided into two groups based on the presence of LV remodelling. Adverse LV remodelling was defined as an absolute increase of LV end-diastolic or end-systolic volumes >20% at 3 months follow-up [21–23]. Analysis of all follow-up data was performed blinded to acute scans.

### Statistical analysis

Statistical analysis was performed using IBM SPSS® Statistics 21.0. Continuous variables are expressed as mean ± SD. Normality for quantitative data was established using the Kolmogorov-Smirnov test. Demographic comparisons were performed with an independent samples t-test. A repeated-measures analysis of variance (ANOVA) was performed on demographic and CMR parameters. Post-hoc univariate analysis was performed by using Tukey test [24]. Step-wise multivariate linear regression was used for parameters with statistical significance]from one-way analysis (p < 0.1). The accuracy of myocardial deformation parameters in predicting presence of MVO or IMH was examined using receiver-operator characteristic (ROC) curve analyses, using Medcalc (v15.8). All statistical tests were 2-tailed; p values < 0.05 were considered significant. To reduce transfer bias, baseline demographics and CMR parameters of the followed up patients were compared to patients who did not receive follow-up CMR by ANOVA.

## Results

Forty-three acute STEMI patients met the inclusion criteria. Demographics of patients and ten healthy volunteers are shown in Table 1. Infarct characteristics on CMR are listed in Table 2. No gender and age based differences in characteristics were present between patient groups (p > 0.1).

**Table 1:** Study demographics

	STEMI with MVO or IMH	STEMI without MVO or IMH	HV	p value
N	25	18	10	–
Age (years)	59 ± 12	57 ± 10	62 ± 9	0.86^*^/0.30^†^
Male	22 (88%)	14 (78%)	3 (30%)	0.69^*^/0.35^†^
Body mass index (kg/m^2^)	29 ± 3	27 ± 3	27 ± 5	0.03^*^/0.28^†^
Current smoker	14 (32%)	9 (21%)	0	0.90^*^
Hypertension	7 (16%)	4(9%)	0	0.88^*^
Hypercholesterolemia	8 (18%)	5 (12%)	0	0.94^*^
Diabetes mellitus	5 (12%)	1(2%)	0	0.30^*^
Pain to balloon time (min)	286 ± 211	376 ± 386	NA	0.33^*^
TIMI flow grade 0/1 pre-PCI	22 (51%)	17 (39%)	NA	0.78^*^
TIMI flow grade 3 post PCI	23 (53%)	18 (42%)	NA	0.28^*^
Peak troponin I >30,000 ng/L	14 (32%)	24 (56%)	NA	0.17
Anterior infarct	12 (28%)	8 (18%)	NA	0.82^*^
Inferior infarct	10 (23%)	7 (16%)	NA	0.94^*^
Lateral infarct	3 (7%)	3 (7%)	NA	0.67^*^

Data as mean ± SD or n(%) unless indicated

*HV* healthy volunteers, *NA* not-applicable, *STEMI* ST-elevation myocardial infarction

^†^p-value between first–second combined versus healthy volunteers

^*^p*-*value between first and second STEMI group

**Table 2:** Imaging parameters at baseline

Characteristic	MI (n = 43)	Healthy volunteers (n = 10)	P value
Ejection fraction (%)	48 ± 10	63 ± 4	<0.001
LV EDVi (ml/m^2^)	82 ± 16	78 ± 20	0.47
LV ESVi (ml/m^2^)	42 ± 12	28 ± 8	<0.001
LV stroke volume (ml)	40 ± 11	49 ± 12	0.023
LGE infarct volume (ml)	15 ± 12	NA	NA
LGE MVO volume (ml)	3 ± 5	NA	NA
GRS (%)	25 ± 8	38 ± 7	<0.001
GRSR (%/s)	164 ± 50	268 ± 125	<0.001
GCS (%)	−13 ± 4	− 20 ± 2	<0.001
GCSR (%/s)	−106 ± 132	− 107 ± 12	0.99
GLS (%)	−13 ± 4	− 20 ± 2	<0.001
GLSR (%/s)	−128 ± 314	− 88 ± 13	0.68

Data as mean ± SD. LV measurements are indexed to body surface area; infarct volumes are unindexed

*LV EDVi* left ventricular end diastolic volume (indexed), *LV ESVi* left ventricular end systolic volume (indexed), *GCS* peak global circumferential strain, *GCSR* peak global circumferential strain rate, *GLS* peak global longitudinal strain, *GLSR* peak global longitudinal strain rate, *GRS* peak global radial strain, *GRSR* peak global radial strain rate

### Baseline data

**Fig. 3:**
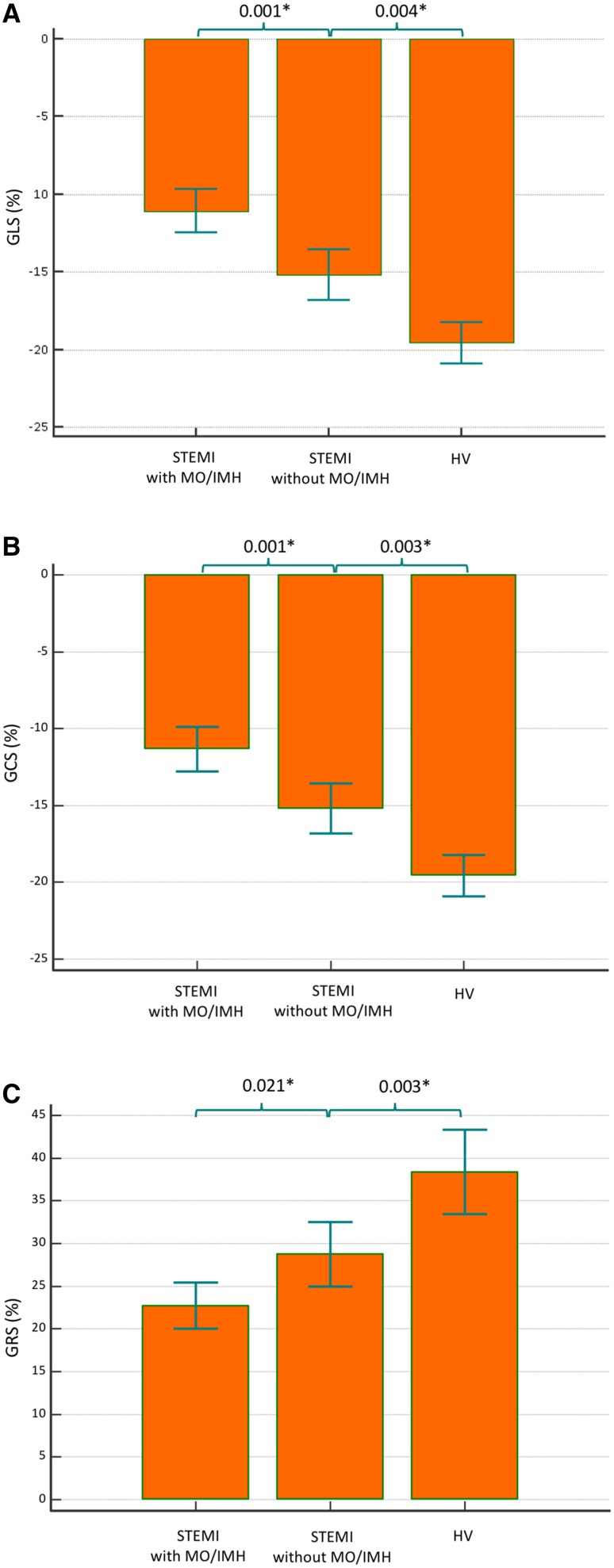
Multiple comparison *bars* of myocardial strain in the study population (whiskers: standard deviations; SD)

Left ventricular EF, left ventricular end-systolic volume (LVESV), GLS, GCS, GRS and GRSR were significantly altered in infarct patients versus healthy volunteers (p < 0.001 for all parameters individually) (Fig. 3). Stroke volume was also reduced in the infarct subjects (p = 0.023 versus controls). Among the 43 infarct patients, 25 patients (58%) had MVO and 24 patients (56%) had confirmed IMH. GRS was significantly lower in patients with MVO or IMH than those without (22.7 ± 7% vs. 29 ± 7%; p = 0.02). Additionally, both GCS and GLS were significantly lower in patients with compared with those without MVO or IMH (GCS: −11.6 ± 3% vs. −15.6 ± 3%, p < 0.001, GLS: −11 ± 3% vs. −15.2 ± 3.3%, p < 0.001) (Fig. 3).

On linear regression analysis, using all the demographics and imaging variables including infarct size, GLS demonstrated the strongest association with presence of MVO or IMH (beta = 0.53, p < 0.001) (Table 3). Additionally, GCS demonstrated stronger correlation to the volume of MVO than GLS (r = 0.57, p < 0.001 vs r = 0.46, p = 0.002) (Table 4). The area under the curve (AUC) for the diagnostic performance of determining the presence of MVO or IMH by GLS was 0.82 (95% CI: 0.69–0.94; p < 0.001). The optimal cut-off value determined by Youden index for GLS was −13.7% for the presence of MVO or IMH (sensitivity 76% and specificity 78%) [25].

**Table 3:** Univariate and multivariate analysis of longitudinal parameters of LV function to CMR derived clinical and prognostic markers

	Microvascular obstruction and intra-myocardial haemorrhage			
	Univariate		Multi-variate (Stepwise)	
	beta	p-value	beta	p-value
Demographics				
Age	0.07	0.62		
Sex	0.13	0.38		
Smoking	0.06	0.70		
Hypertension	0.07	0.67		
Hypercholesterolemia	0.05	0.77		
Diabetes mellitus	0.21	0.19		
Pain-balloon time	−0.15	0.33		
CMR parameters				
LVEDVi	0.09	0.57		
LVESVi	0.38	0.01*	0.17	0.26
EF	−0.50	0.001*	−0.27	0.13
GRS	−0.39	0.01*	−0.07	0.67
Infarct size	0.50	0.001*	0.36	0.01*
GCS	0.52	<0.001*	0.29	0.16
GLS	0.53	<0.001*	0.53	<0.001**
GRSR	−0.24	0.122		
GCSR	−0.12	0.44		
GLSR	0.18	0.26		

*EF* ejection fraction, *LVEDVi* left ventricular end-diastolic volume indexed, *LVESVi* left ventricular end-systolic volume indexed, *GCS* peak global circumferential strain, *GCSR* peak global circumferential strain rate, *GLS* peak global longitudinal strain, *GLSR* peak global longitudinal strain rate, *GRS* peak global radial strain, *GRSR* peak global radial strain rate

*Significant p-value

**Most significant p-value in multivariate

**Table 4:** Association of baseline CMR volumetric and strain parameters to size of microvascular obstruction

	Location of infarct		Infarct volume (%)		Microvascular obstruction volume (%)	
	r	p value	r	p value	r	p value
EF	0.29	0.06	−0.37	0.01	−0.37	0.02
LVEDVi	−0.18	0.24	0.20	0.20	0.08	0.60
LVESVi	−0.24	0.12	0.41	0.01	0.30	0.05
SVi	0.03	0.87	−0.19	0.23	−0.24	0.13
GRS	0.19	0.21	−0.32	0.03	−0.39	0.01
GRSR	0.04	0.79	−0.24	0.13	−0.13	0.41
GCS	−0.18	0.25	**0.54**	<**0.001**	**0.57**	<**0.001**
GCSR	0.21	0.18	−0.01	0.94	− 0.30	0.06
GLS	−**0.33**	**0.03**	0.34	0.02	0.46	0.002
GLSR	0.12	0.44	0.20	0.20	0.10	0.52

*EF* ejection fraction, *GCS* peak global circumferential strain, *GCSR* peak global circumferential strain rate, *GLS* peak global longitudinal strain, *GLSR* peak global longitudinal strain rate, *GRS* peak global radial strain, *GRSR* peak global radial strain rate, *LVEDVi* left ventricular end-diastolic volume indexed, *LVESVi* left ventricular end-systolic volume indexed; *r* Pearson correlation coefficient

Bold text represents most signifcant r value and it’s corresponding p-value

### Follow-up data

Thirty-four of the 43 patients underwent 3 month follow up CMR; six patients declined further follow-up and in three patients the scan quality of cines on follow up was not suitable for FT analysis. Demographics parameters (age, gender, hypertension, hypercholesterolaemia, smoking history, diabetes mellitus) and baseline CMR parameters (presence of MVO or IMH, LVEDVi, LVESVi and all strain parameters) were not significantly different in the nine patients who did not attend for follow-up scans compared with the overall study population (p > 0.1). All 34 follow up scans showed complete resolution of MVO and IMH. As compared to baseline, relative improvement in EF was 19 ± 24.5%. Of all the baseline CMR parameters (LVEDVi, LVESVi, GLS, GCS, GRS, MVO, IMH), LVESVi (r = 0.99, p < 0.002) and GLS (r = 0.97, p < 0.006) demonstrated the strongest correlation with improvement in EF at follow-up scan. GCS (r = 0.95, p = 0.01) and GRS (r = 0.91, p = 0.02) also demonstrated good correlations with improvement in EF at follow-up.

### Adverse LV Remodelling

Out of 34 patients with follow-up data, 6 (17%) patients demonstrated adverse left ventricular remodelling. From all CMR baseline parameters, GLS demonstrated the strongest diagnostic performance in predicting adverse LV remodelling (AUC = 0.79; 95% CI 0.60–0.98; p = 0.03) (Table 5).

**Table 5:** Association of baseline CMR parameters to adverse LV remodelling at follow-up visit

	Adverse LV remodelling
LVEDVi	AUC = 0.60; 95% CI 0.34–0.86; p = 0.44
LVESVi	AUC = 0.60; 95% CI 0.32–0.87; p = 0.47
LV EF	AUC = 0.26; 95% CI 0.00–0.52; p = 0.07
GLS	AUC = 0.79; 95% CI 0.60–0.98; p = 0.03*
GLSR	AUC = 0.68; 95% CI 0.42–0.95; p = 0.16
GRS	AUC = 0.32; 95% CI 0.11–0.54; p = 0.18
GRSR	AUC = 0.34; 95% CI 0.16–0.52; p = 0.22
GCS	AUC = 0.71; 95% CI 0.48–0.87; p = 0.11
GCSR	AUC = 0.57; 95% CI 0.35–0.78; p = 0.62

*AUC* area under the curve, *CI* confidence interval, *EF* ejection fraction, *GCS* peak global circumferential strain, *GCSR* peak global circumferential strain rate, *GLS* peak global longitudinal strain, *GLSR* peak global longitudinal strain rate, *GRS* peak global radial strain, *GRSR* peak global radial strain rate, *LV* left ventricle, *LVEDVI* left ventricular end-diastolic volume indexed, *LVESVI* left ventricular end-systolic volume indexed,*r* Pearson correlation coefficient

## Discussion

The main findings of this study are as follows: first, myocardial deformation imaging by CMR reliably detects changes in acute infarct patients versus healthy controls. Second, the presence of MVO or IMH in acute reperfused STEMI is most strongly associated with GLS. Third, GLS showed modest association with adverse LV remodelling.

Our data complement the results of several previous investigations of the role of CMR-derived strain imaging in reperfused STEMI patients [18, 26–28]. Kidambi et al. studied the role of myocardial deformation using tissue tagging derived strain in an acute reperfused infarct population [18]. They demonstrated that regional functional recovery is poor in myocardial segments with MVO and IMH. Wong et al. demonstrated that circumferential strain (CS) using tissue tagging correlates better than circumferential strain rate with regional functional recovery [29]. Both of these studies used tissue tagging, which has a relatively low temporal resolution (<30 frames/s), potentially limiting its accuracy, especially in patients with higher heart rates. Moreover, acquisition of tissue tagged images often requires long series of breath holds, and tag fading during diastole limits the assessment of myocardial relaxation. FT analysis of cine loops may overcome these limitations. A study by Khan et al. compared tissue tagging to FT-derived strain in 24 acute reperfused STEMI patients. FT-derived strain was quicker to analyse, tracked the myocardium better, had better inter-observer variability and stronger correlations with infarct and oedema [27].

In a study of 74 patients, Buss et al. demonstrated that FT-derived GCS is strongly associated with infarct size and trans-murality of scar on LGE imaging [28]. This study also demonstrated that FT-derived GCS was more accurate than GLS for predicting preserved LV function at follow-up. Notably, this study did not evaluate LV remodelling, presence of MVO, presence of IMH or functional recovery of LV defined by improvement in EF. Additionally, in this study, the FT-derived strain analysis algorithm tracked only the endo-/epi-myocardium to compute strain, and did not track pixels within the myocardium [30]. Tracking pixels within the myocardium is important, especially in the setting of acute reperfused infarct where each layer of myocardium (endo-, mid- and epi-) is going through different pathophysiological processes.

Our study adds to the growing body of literature on the ability of CMR to quantify left ventricular deformation with FT. We have shown that FT-derived myocardial deformation parameters (GCS, GRS and GLS) are altered significantly in patients with MVO or IMH (p < 0.05). MVO and IMH affect predominantly the sub-endocardium, where most of the longitudinal myocardial fibres are located. It is thus plausible that GLS is the strongest predictor of MVO and IMH as shown in our study. GLS also demonstrated modest diagnostic performance to predict adverse LV remodelling at follow-up more than any other deformation parameter. In this study, the volume of MVO and infarct size were more strongly associated with GCS than GLS (Table 4). These results are not unexpected as larger infarcts with MVO will involve more myocardium transmurally.

### Role of echocardiography

It is acknowledged that strain examination is more readily available by echocardiography than CMR. All modern echocardiographic systems come with strain packages [31, 32]. Early changes of microvascular obstruction (MVO) after AMI have been demonstrated by contrast echocardiography [33–35]. In patients with AMI, echocardiographic studies can be performed at the bedside and GLS assessment may be used as a ‘gatekeeper’ for further advanced imaging, for example, multi-parametric tissue characterization on CMR. Further studies are needed to explore how echocardiography derived strain parameters compare to CMR-FT derived strain.

### Clinical implications

Our findings have possible clinical implications as FT-strain analysis can be performed rapidly from standard cine CMR images and allows the detection of the functional effects of MVO and IMH without the need for additional CMR tissue characterisation techniques (T2W and T2*) and analysis methods. From our one-center experience, the time for total left ventricular strain analysis by CMR FT is approximately 7 min. As demonstrated, a cut off value of −13.7% for GLS detects MVO or IMH with a sensitivity of 76% and specificity of 77.8%. GLS can potentially predict the presence of MVO or IMH early after PPCI for STEMI. MVO and IMH are independent histopathological and cardiac imaging markers of adverse prognosis and we speculate that their early detection from routinely acquired CMR cines may help tailor appropriate pharmacological interventions or guide stem cell therapy. Patients with known allergy to gadolinium-based contrast agents or patients with end-stage renal failure may also benefit from this technique.

### Study limitations

In this study, we excluded patients who were unstable post-PPCI (higher Killip class, not able to lie flat because of shortness of breath and use of invasive monitoring). These patients are more likely to represent a higher risk group with an adverse prognosis. In our study population, the majority of patients with MVO had IMH and only one patient with MVO had no IMH. Hence, the data on GCS for IMH detection should be interpreted with caution.

Another important limitation of our study was that 9 of 43 patients did not have follow-up CMR scans. This may have introduced transfer bias although the two groups were not different for demographic and standard CMR parameters.

In our study, at follow-up, only 6 (17%) patients had adverse LV remodelling and hence the demonstrated diagnostic performance of GLS to predict remodelling should be interpreted with caution.

In the present study, only global parameters of strain were investigated. Assessment of regional left ventricular strain parameters by CMR FT demonstrates regional variations and their clinical role remains very speculative [36].

## Conclusions

Myocardial deformation changes adversely in patients with acute STEMI. Baseline GLS by FT-analysis of cine CMR is strongly associated with the presence of MVO or IMH and could be used as surrogate functional imaging marker of these acute myocardial pathological changes in patients with acute STEMI. Baseline GLS demonstrated stronger association with adverse LV remodelling than other CMR parameters.

## Abbreviations

AAR: Area at risk
AMI: Acute myocardial infarction
AUC: Area under the curve
CMR: Cardiovascular magnetic resonance
EF: Ejection fraction
FT: Feature tracking
GCS: Peak global circumferential strain
GCSR: Peak global circumferential strain rate
GLS: Peak global longitudinal strain
GLSR: Peak global longitudinal strain rate
GRS: Peak global radial strain
GRSR: Peak global radial strain rate
IMH: Intramyocardial haemorrhage
LGE: Late gadolinium enhancement
LV: Left ventricle
LVEDVi: Left ventricular end diastolic volume indexed
LVESVi: Left ventricular end systolic volume indexed
MR: Magnetic resonance
MVO: Microvascular obstruction
PCI: Percutaneous coronary intervention
RF: Radiofrequency
ROC: Receiver operator characteristics
SD: Standard deviation
STEMI: ST-elevation myocardial infarction
T2*: T2-star-weighted imaging
T2W: T2-weighted imaging
